# From injury prevention to standardized scoring: a bibliometric analysis of thematic evolution and emerging trends in the development of the breakdance competition system (1984–2026)

**DOI:** 10.3389/fspor.2026.1901612

**Published:** 2026-07-28

**Authors:** Hang Yin, Xi Ling

**Affiliations:** 1 Faculty of Education, Thailand National Sport University, Chonburi Campus, Chonburi, Thailand; 2 School of Physical Education, Qujing Normal University, Qujing City, China; 3 Faculty of Education, Silpakorn University, Sanam Chandra Palace Campus, Nakhon Pathom, Thailand

**Keywords:** bibliometric, breakdance, breaking, competition systems, Olympic

## Abstract

When Breaking successfully debuted at the 2024 Paris Olympic Games in France, it attracted strong attention from the academic community toward this emerging competitive sport. Given that Breaking is a newly included Olympic sport that urgently requires development and remains controversial, this review aims to conduct a quantitative analysis of the academic research achievements related to the construction of the Breaking competition system over the past more than 40 years. The researchers selected literature from the Web of Science Core Collection and the Scopus database from 1984 to 2026 as data sources and conducted bibliometric and visualization analyses using CiteSpace, VOSviewer, and R Bibliometrix. Meanwhile, analyses were carried out on core journals, core research teams, research countries, hot keywords, as well as the development process and development trends. The results showed that the research field of the Breaking competition system is developing vigorously. The findings are as follows: (1) The United States leads the world by a large margin in the number of publications, while the United Kingdom and Canada rank next in academic influence; several research groups have formed among the core authors, with Yang, Wyon, and Miura being typical examples. It is worth noting that Sato published the largest number of papers over the longest period of time. Among research institutions, the University of Tokyo in Japan has published the largest number of studies, while *Dance Research Journal* has the greatest influence and the *Journal of Dance Medicine and Science* has the highest output. Among the journal research areas related to this topic, “MEDICINE, MEDICAL, CLINICAL” and “PSYCHOLOGY, EDUCATION, HEALTH” occupy the frontier of research fields, while “NEUROLOGY, SPORT, OPHTHALMOLOGY” have become the most influential research pathways. (2) This field has experienced three stages of development, namely the spontaneous research phase, the initial development phase, and the Olympic-oriented standardization construction phase, mainly focusing on four areas: terminology origins, medical support, rule framework, and competitive psychology and social adaptation. (3) “Case report” is the longest-lasting research hotspot, while “hip hop” has become the most cutting-edge hotspot keyword. Based on existing studies, future research should develop toward three directions: rule quantification, judging consistency, and cultural and competitive synergy. This review provides scholars in the field of Breaking competition system research with a clear knowledge map and offers theoretical support for policymakers of Breaking competitions to formulate more scientific decisions.

## 1 Introduction

Since the International Olympic Committee announced in 2018 that Breaking would be included as a pilot event in the 2018 Summer Youth Olympic Games and later confirmed it as an official competition event for the 2024 Summer Olympics (1, 2), the sport has attracted significant attention from both the public and the academic community. On the Olympic stage, the remarkable technical performances of Breaking athletes captivated audiences while simultaneously drawing extensive scholarly interest. Although Breaking has gained considerable popularity, the public still holds uncertainties regarding the construction of the Breaking competition system, particularly in terms of the training system, selection processes, and competency evaluation.

In fact, scholarly research on Breaking has never ceased. Existing studies cover a wide range of areas, including historical research, sports medicine, and sports training (3–5). Some scholars explore the pedagogical approaches and training methods of Breaking (6, 7). In addition, several studies focus on the positive effects of Breaking on learners' creativity and psychological wellbeing (8, 9). Despite the increasing number of studies related to the Breaking competition system, there remains a lack of systematic research examining the development patterns and trends in this field. For example, in the limited number of existing studies, Breaking is often treated merely as a secondary element within broader research topics, lacking a dedicated breakdance-centered perspective for systematically analyzing this street-based activity. Moreover, a few studies have examined the developmental stages and characteristics of the Breaking competition system (10, 11). Without a systematic understanding of the current status and future trends of this field, competition organizers may confuse Breaking with other forms of sport dance. Furthermore, policymakers may design competition systems in ambiguous or unclear ways, making it difficult for the public to develop an objective understanding of this classic street movement and potentially affecting the sustainable development of Breaking. A typical example is that the International Olympic Committee announced the removal of Breaking from the program of the 2028 Summer Olympics (12).

Under such unfavorable circumstances, promoting the healthy competitive development of Breaking as an Olympic sport has become particularly important. With the advancement of Breaking’s Olympic competitive process, academic research on its competition system has gradually increased. However, current studies have not yet formed a comprehensive analysis of the evolution of the Olympic Breaking competition system, particularly lacking macro-level bibliometric reviews and visualized interpretations. Addressing these limitations through the identification of research hotspots, the analysis of evolutionary logic, and the detection of emerging research frontiers can help fill existing gaps. Such an approach can provide new perspectives and theoretical support for improving and developing the Olympic Breaking competition system, while enabling researchers to accurately understand the evolution of research themes and the structure of knowledge within this field.

This review aims to apply bibliometric methods and visualization tools to analyze literature in the Breaking research field from 1984 to 2026, covering both the pre- and post-Olympic inclusion periods. By doing so, it seeks to identify the developmental patterns and characteristics of the field and to construct a clear framework for the Breaking competition system. The innovation of this review lies in helping scholars gain a comprehensive understanding of the Breaking competition system and providing decision-making support and reference for policymakers responsible for Breaking competitions, thereby promoting the sustainable development of this Olympic sport. Specifically, this review addresses the following research questions:

Research Question 1 (RQ1): What is the distribution of countries, institutions, and authors in the field of Breaking research?

Research Question 2 (RQ2): What academic journals and research fields have been involved in Breaking research over the past four decades?

Research Question 3 (RQ3): What is the developmental trajectory of research themes in Breaking, and which areas are the primary focuses?

Research Question 4 (RQ4): What are the research hotspots and future development trends in Breaking?

## 2 Literature review

### 2.1 The origins of the Breaking competition system

Breaking originated from breakdance and represents one of its primary branches. Breakdance is a street movement that emerged in the 1970s in the Bronx, New York, United States (13). Initially, Breaking served as a medium of self-expression for African American and Puerto Rican youth, with its core competitive form being the battle (14). Outside the competitive arena, the cypher functions as a common space for everyday interaction among breakdancers (15). In 1984, the film Breakin' brought breakdance into the public spotlight and sparked widespread enthusiasm among young people (16, 17). During its long period of spontaneous development, Breaking existed largely outside the formal sports system, forming a unique non-institutionalized competitive ecology (18), until it gradually began transitioning toward an official competition system after 2018. Precisely because of this organically evolving state, the public often holds inconsistent understandings of terms such as Breaking, breakdance, hip-hop dance, and street dance (19), which to some extent has also influenced the healthy development of the Olympic competition system.

### 2.2 Research progress in Breaking competition systems

The Olympic competitive transformation of Breaking essentially represents a process in which its competition system gradually shifts from informal community exchange to professionalized competition structures. Taking the pilot event at the 2018 Summer Youth Olympic Games as a critical turning point (20), the evolution of Breaking competitions can be clearly divided into two stages: the pre-Olympic underground competition stage and the post-Olympic standardized competition stage, each demonstrating distinctive competitive characteristics.

Specifically, prior to the official announcement in 2018 that Breaking would be included in the Youth Olympic Games, most Breaking competitions were organized as spontaneous underground events, forming a competitive ecology rooted in street culture and lacking unified regulations. During this stage, Breaking competitions were sometimes labeled as street dance or hip-hop dance events (21, 22), and not all competitions explicitly identified themselves as Breaking events. Traditional studies on the Breaking competition system mainly focus on three aspects.

1. Research on Breaking terminology. In many studies, Breaking is frequently confused with street dance or hip-hop dance. For example (23), classified hip-hop as a branch of street dance, while Chew and Mo (8) regarded Breaking and hip-hop dance as forms of street dance. Although hip-hop scholar Ling (19) called for the standardization of Breaking terminology, the academic usage of the term remains inconsistent.
2. Research on the evaluation of Breaking. Sato et al. was the first scholar to propose research on competition evaluation; in his work, Breaking was included within the broader category of hip-hop dance contests, with no clear distinction made between breakdancers and hip-hop dancers (22). Subsequently, hip-hop scholars (24) designed an assessment of breakdancers' knowledge, providing a reference for the standardization of Breaking evaluations. Nevertheless, these studies lack a holistic examination of the Olympic scoring criteria for Breaking, and their research designs lack empirical evidence, remaining at the level of advocacy and preliminary conceptualization (25).
3. Research on injuries among breakdancers. Although some scholars have examined injuries among breakdancers using cross-sectional surveys and retrospective analyses (26, 27), concerns remain regarding the representativeness of the samples. Existing research largely focuses on common musculoskeletal injuries, while relatively little attention has been paid to the pathological mechanisms, risk factors, and prevention strategies for discipline-specific injuries. Moreover, these studies have not sufficiently addressed new injury risks arising from changes in training modes under Olympic competitive conditions (28). It is evident that current research suffers from a clear shortcoming: the findings are not sufficiently integrated with competitive training practices, and a comprehensive injury prevention and rehabilitation system has yet to be established.

## 3 Methodology and materials

The research team adopted a bibliometric analysis approach to conduct a quantitative analysis of literature related to the Breaking competition system. Bibliometric analysis, as a scientific research paradigm, primarily relies on visualization and statistical tools to systematically analyze bibliometric data (29). From a functional perspective, this method can cover a wider range of research topics and dimensions. It not only enables researchers to efficiently process large-scale research data but also helps reveal the underlying patterns and structural characteristics within a target research field (30, 31).

This review strictly followed the guidelines of Preferred Reporting Items for Systematic Reviews and Meta-Analyses (PRISMA) to ensure the quality, rigor, and reproducibility of the research (32). On this basis, a comprehensive literature search and screening strategy was established, including the design of search keywords as well as the criteria for literature inclusion and exclusion (33).

### 3.1 Data collection

The research team selected literature from the Web of Science Core Collection (WoSCC) and the Scopus database as the primary data sources. Both databases are widely recognized as high-quality sources for bibliometric analysis, as they compile important academic achievements in the field of social sciences and demonstrate high levels of reliability, validity, and relevance (34, 35). Among them, Scopus is operated and managed by Elsevier and serves as a comprehensive scientific database covering multidisciplinary research outputs. The WoSCC is maintained by Clarivate and provides access to multiple sub-databases. Both databases support integrated retrieval of different types of research outputs, including articles, book chapters, and conference papers, and provide multiple citation export formats. To ensure the robustness, accuracy, and validity of the study, unified inclusion and exclusion criteria were applied across both databases. The detailed criteria are presented in Table 1.

**Table 1:** Inclusion and exclusion criteria.

Type	Inclusion and exclusion criteria
Research Content Standards	(1) Included literature must contain clearly defined research questions, methodologies, and findings
	(2) This review primarily focused on core research themes related to the Breaking competition system, particularly in B-boy, street dance, breakdance, and hip-hop dance
Literature Quality Standards	(1) Exclude literature with fewer than five pages
	(2) Remove literature lacking key information, including abstract, author(s), keyword fields, and reference list
	(3) Remove retracted literature
	(4) Remove literature published without a rigorous double-blind peer review process, such as conference papers, editorials, or brief introductions

### 3.2 Search strategy

During the design of the search strategy, the research team optimized the search terms based on previous studies by Yang and Whatman (36) and Ling (19), while aligning them with the research questions of this review. A comprehensive literature search was conducted in both the WoSCC and Scopus databases. After manually removing irrelevant records, a total of 150 valid publications from WoSCC and 199 valid publications from Scopus were obtained. The literature search was conducted on 13 February 2026. Therefore, only publications indexed in WoSCC and Scopus on or before that date were included. To ensure data completeness, the full records from WoSCC, including citation information, were exported in plain text format, while the full citation records from Scopus were exported in CSV format.

WoSCC and Scopus were selected because they are the two most commonly used comprehensive, multidisciplinary databases in bibliometric research. Previous methodological studies have shown that combining WoSCC and Scopus provides broad coverage of peer-reviewed literature while minimizing database-specific biases. Other databases such as PubMed or Embase focus primarily on biomedical literature and have significant overlap with WoSCC and Scopus; Google Scholar, on the other hand, includes non-peer-reviewed sources, which may compromise the consistency of data in bibliometric analyses. The detailed search and screening procedures are presented in Table 2.

**Table 2:** Search strategy.

Item	Web of Science Core Collection (WoSCC)	Scopus
Database	Web of Science Core Collection (WoSCC)	Scopus
Search field	Topic (TS)	TITLE-ABS-KEY
Complete search strategy	TS = (“Street dance” OR “streetdance” OR “street-dance” OR “Street Dance” OR “street dances” OR “streetdancing” OR “street dancing” OR “hiphop dance” OR “hip-hop dance” OR “Hiphop dance” OR “Hip-Hop dance” OR “HipHop dance” OR “breakin`” OR “break-dance” OR “breakdance” OR “break dance” OR “breakdancing” OR “breaking dance” OR “breakdancer” OR “breakdancers” OR “b-girls” OR “b-boys” OR “b-boying” OR “b-boy” OR “b-girl” OR “piliwu” OR “female breakdancer” OR “jiewu” OR “break-dance” OR “Hiphop dancer” OR “Hip-Hop dancer”)	TITLE-ABS-KEY = (“Street dance” OR “streetdance” OR “street-dance” OR “Street Dance” OR “street dances” OR “streetdancing” OR “street dancing” OR “hiphop dance” OR “hip-hop dance” OR “Hiphop dance” OR “Hip-Hop dance” OR “HipHop dance” OR “breakin`” OR “break-dance” OR “breakdance” OR “break dance” OR “breakdancing” OR “breaking dance” OR “breakdancer” OR “breakdancers” OR “b-girls” OR “b-boys” OR “b-boying” OR “b-boy” OR “b-girl” OR “piliwu” OR “female breakdancer” OR “jiewu” OR “break-dance” OR “Hiphop dancer” OR “Hip-Hop dancer”)
Search date	1 January 1984—13 February 2026	
Language restriction	English	
Document types	Articles and Review Articles	Articles and Review Articles
Indexes searched	SCI-Expanded, SSCI, ESCI, A&HCI	All indexed journals available in Scopus
Initial records retrieved	430	620
Duplicate removal	Records were merged using Citespace and duplicate publications were removed automatically, followed by manual verification based on title, author(s), publication year, journal, and DOI	
Screening procedure	Literature screening followed the PRISMA 2020 guidelines. Titles, abstracts, and bibliographic information were independently checked against the predefined inclusion and exclusion criteria	
Eligibility assessment	Full records were examined to determine their relevance to the research scope after duplicate removal and preliminary screening	
Export format	Plain Text (.txt)	CSV/BibTeX
Bibliometric software	CiteSpace, VOSviewer, and Bibliometrix (R package)	
Reproducibility statement	The complete search strategy, screening process, duplicate removal procedure, and data processing workflow are reported to facilitate transparency and reproducibility	

It should be noted that in this review, the “Breaking competition system” is defined as a comprehensive framework that supports organization, regulation, evaluation, governance, athlete training, officiating, performance assessment, and the sustainable development of Breaking competition. Therefore, the scope of this bibliometric analysis is not limited to competition rules or refereeing systems but also includes discussions on sports injury prevention, biomechanics, psychology, athletic performance, dance pedagogy, and athlete development, as these fields provide the scientific foundation for establishing fair, safe, standardized, and internationally recognized competitive practices.

### 3.3 Data standardization

To avoid potential biases caused by inconsistent terminology in bibliometric analysis (37), this review conducted data disambiguation on the raw data exported from WoSCC prior to formal analysis. The raw dataset contained multiple expressions referring to the same concept, and data disambiguation is a critical prerequisite for ensuring the precision and reliability of bibliometric results (38, 39). Specifically, following the data standardization procedure proposed by Taskin and Al (40), the study first merged synonymous keywords and author-related information (e.g., “breakdance,” “break dance,” and “breakdancing”). Second, the uniqueness of institutional and journal names was verified, and inconsistencies such as journal abbreviations or name changes were systematically standardized.

### 3.4 Literature screening

**Figure 1:**
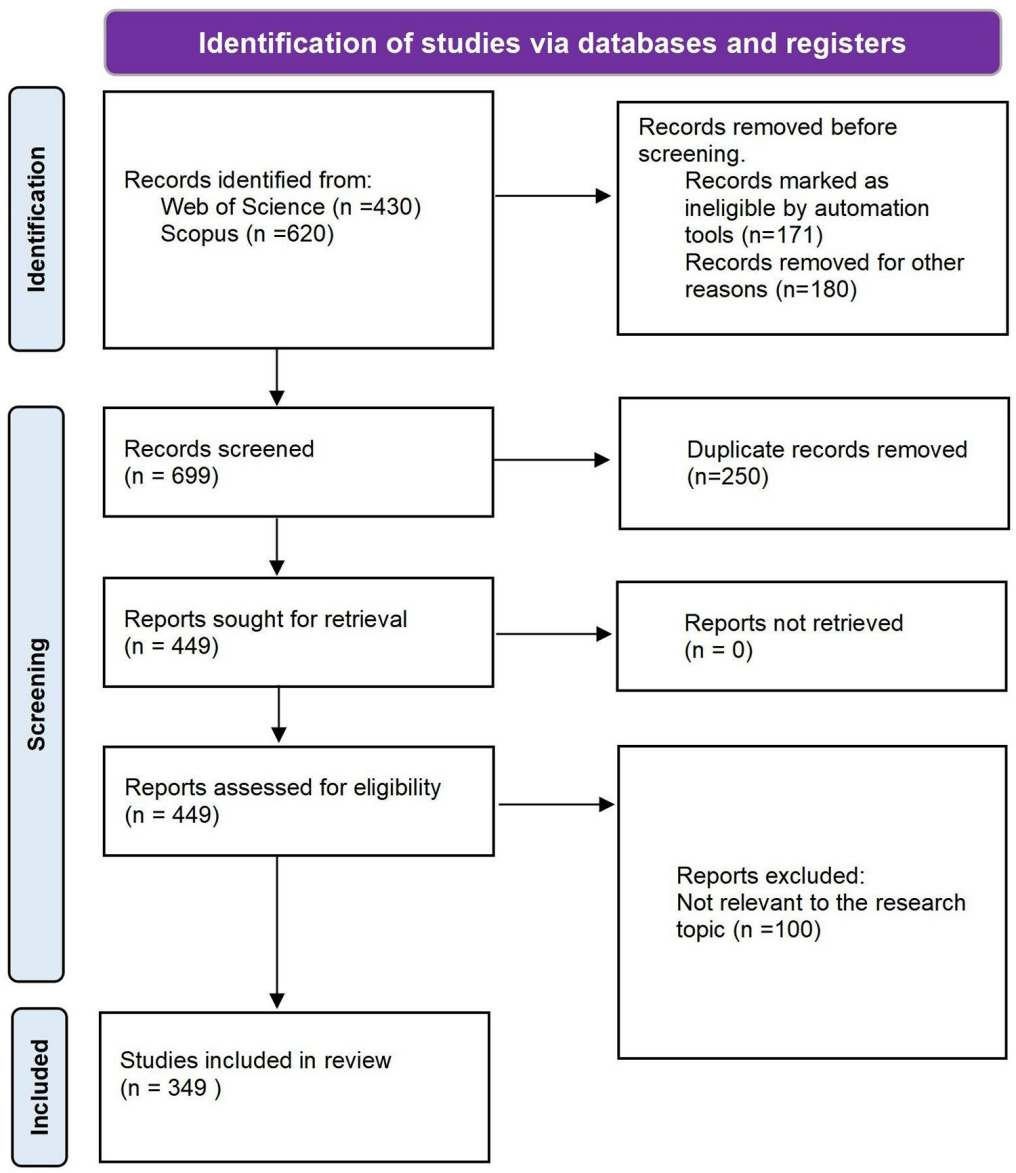
A literature screening flowchart based on the PRISMA guidelines.

The research team initially retrieved 1,050 publications from the WoSCC and Scopus databases. In addition to limiting document types to articles and reviews, other document types such as retracted papers and conference proceedings were removed. After manual screening, a total of 349 valid publications were retained for further analysis. The screening process conducted according to the PRISMA guidelines is illustrated in Figure 1.

## 4 Bibliometric results and analysis

### 4.1 Descriptive statistics

**Figure 2:**
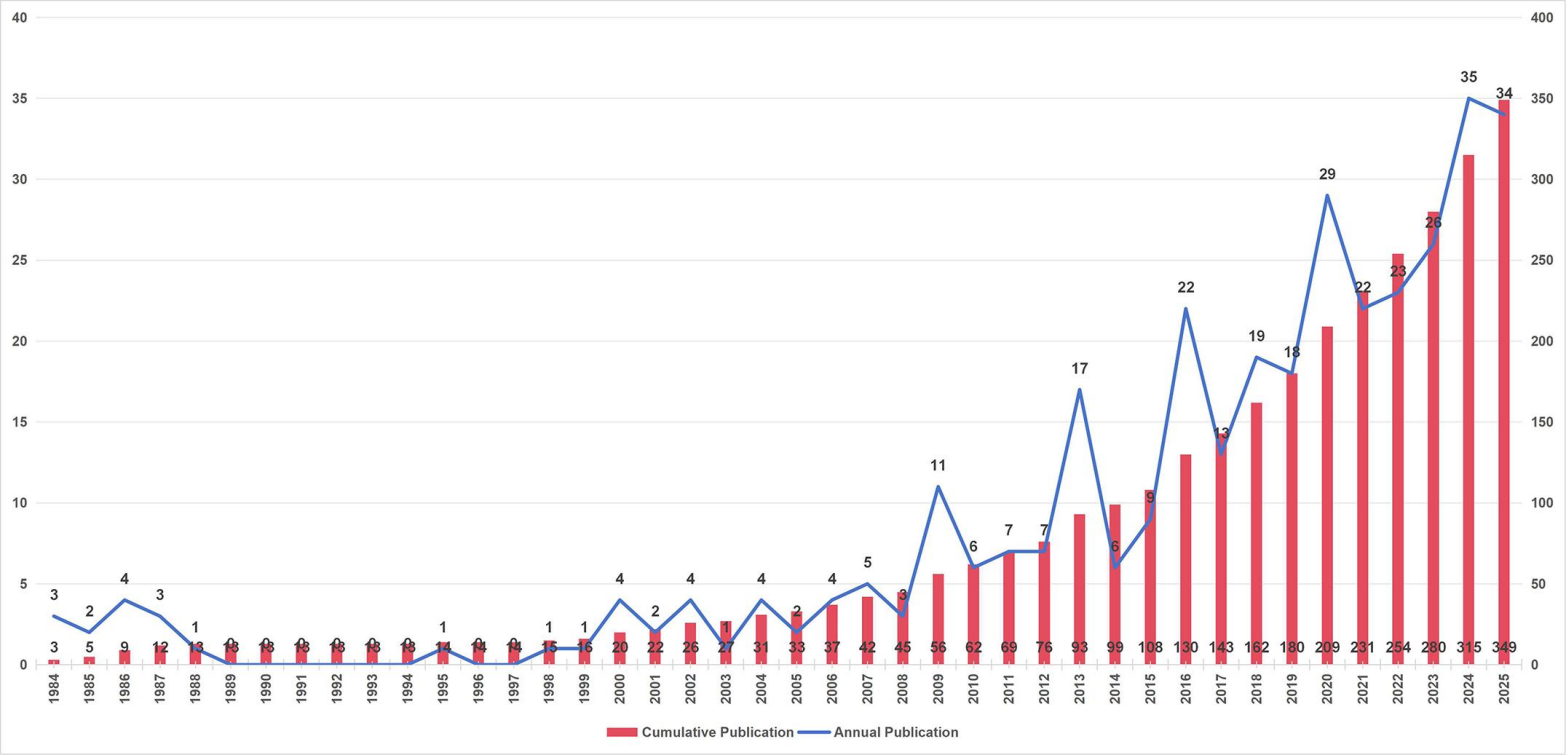
Annual and cumulative number of publications in the field of the Breaking competition system worldwide from 1984 to 2026.

A total of 349 publications related to the Breaking competition system were collected worldwide in this review, covering the period from 1 January 1984 to 13 February 2026. Since 2,000, the number of publications in this field has increased steadily. In particular, 2024 reached 35 publications, representing the highest number recorded in history. Following the Olympic inclusion of Breaking, research in this field has entered a period of rapid growth. Figure 2 illustrates the overall development trend of global publications related to the Breaking competition system.

As shown in Figure 2, the development of research on the Breaking competition system can be divided into two stages: the pre-Olympic stage and the post-Olympic stage. The first phase (pre-Olympic) spanned from 1984 to 2017. Although Breaking was not yet an official Olympic sport during this phase, it had already attracted attention from the academic community. The second phase (post-Olympic) runs from early 2018 to early 2026; following its inclusion in the Olympics, the number of publications in the field of research on the Breaking competition system has grown rapidly. In addition, as an Olympic sport capable of attracting younger audiences, Breaking has gained broad recognition within the global academic community for the research value of its competition system (41). The growth of commercial collaborations and international competitions has further strengthened the practical orientation and real-world significance of related studies, jointly contributing to the rapid increase in publication output.

### 4.2 Quantitative analysis of countries/regions

**Figure 3:**
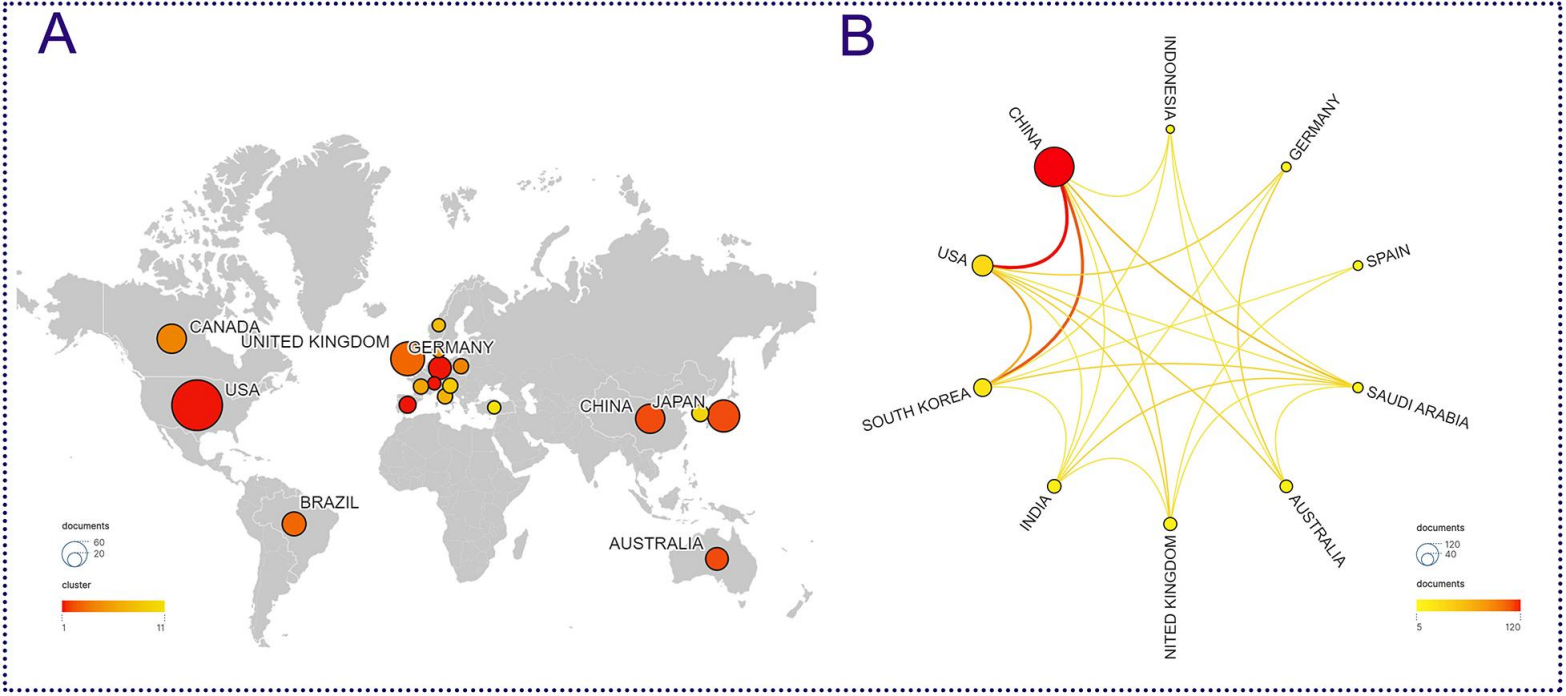
**(A)** Visualization of the number of publications by the top ten countries in the field of the Breaking competition system. **(B)** Academic collaboration networks among countries.

A total of 139 countries and regions have published research related to the Breaking competition system. The top 10 countries/regions are presented in Table 3. Figure 3A visually illustrates the research contributions of different countries in this field, while Figure 3B presents the details of scientific collaboration among countries worldwide.

**Table 3:** Top 10 countries by publication contributions in the field of Breaking competition systems.

Rank	Country/region	Count	Citations	Percentage	Average citations per article
1	United States	44	1,954	12.61	44.41
2	United Kingdom	20	296	5.73	14.8
3	Japan	18	194	5.16	10.78
4	China	15	110	4.3	7.33
5	Canada	15	203	4.3	13.53
6	Brazil	10	94	2.87	9.4
7	Germany	9	146	2.58	16.22
8	Australia	9	76	2.58	8.44
9	Spain	5	22	1.43	4.4
10	South Korea	5	13	1.43	2.6

By combining Table 3 and Figure 3A, it can be observed that the United States overwhelmingly leads in both publication output and academic influence. This pattern closely corresponds to the country’s status as the birthplace of Breaking culture. The United Kingdom and Japan follow closely in terms of the number of publications. Notably, Germany’s papers have received the highest number of citations. Overall, global research efforts are highly concentrated in a few countries, reflecting disparities in the popularity and competitive development of Breaking across different regions, as well as differences in the level of attention various countries devote to research in this field within the context of the sport's institutionalization in the Olympic Games.

An analysis of Figure 3B reveals that international academic collaboration in the field of Breaking competition systems is close-knit. China occupies a central hub position, engaging in intensive collaboration with the United States and South Korea, while countries such as Indonesia and Saudi Arabia are situated at the periphery of the network. Overall, the structure exhibits a hierarchical pattern characterized by core dominance and peripheral supplementation. Nevertheless, cooperation among other countries remains relatively limited, and significant disparities persist in research productivity, collaboration depth, and breadth across regions. Strengthening international collaboration will therefore be essential for promoting global coordination and improving the overall quality of research on the Olympic development of Breaking.

### 4.3 Quantitative analysis of institutes

In the global field of Breaking competition system research, the top 10 institutions have collectively produced 38 publications, accounting for approximately 10.9% of the total global output. Among them, the University of Tokyo ranks first worldwide in both publication output and average citations per article, with six publications and an average of 19.17 citations per paper, making it the most authoritative research institution in this field. Nagoya Gakuin University and the University of Wolverhampton are tied for second place. Although each institution has produced five publications, their average citation rates are significantly lower than that of the University of Tokyo, reflecting a pattern of high publication volume but relatively low citation impact. In contrast, the citation performance of other institutions remains relatively balanced. Detailed information is presented in Table 4.

**Table 4:** Top 10 institutes by publication contributions for the Breaking competition system.

Rank	Organization	Documents	Citations	Percentage	Average citation per paper
1	University of Tokyo	6	115	1.72	19.17
2	Nagoya Gakuin University	5	41	1.43	8.2
3	University of Wolverhampton	5	66	1.43	13.2
4	Universidade Federal do Rio Grande do Sul	4	21	1.15	5.25
5	Aichi Shukutoku University	3	47	0.86	15.67
6	Fukuoka University	3	47	0.86	15.67
7	Japan Society for the Promotion of Science	3	58	0.86	19.33
8	National Institute of Dance Medicine and Science	3	46	0.86	15.33
9	University of Manitoba	3	51	0.86	17
10	University of Ottawa	3	51	0.86	17

As given in Table 4, the field of research on Breaking competition systems is dominated by Japanese institutions, with institutions from Canada, the United Kingdom, Brazil, and other countries contributing a diverse array of research. Research efforts in this field are highly concentrated in developed countries, primarily in Asia, North America, and Europe, resulting in uneven regional development. This pattern reflects the varying degrees of emphasis placed by different countries on Breaking research within the context of the sport's institutionalization in the Olympic Games. It is worth noting that research investment by academic institutions in various countries does not correlate directly with competition results. For example, although China won the bronze medal in the women’s Breaking event at the 2024 Summer Olympics (42), no Chinese institution appears among the world's top 10 research institutions, suggesting that China still has considerable room for development in this research area. In addition, from the perspective of institutional types, the high citation impact of organizations such as the Japan Society for the Promotion of Science and the National Institute of Dance Medicine and Science indicates that research in this field tends to focus on areas such as sports medicine, injury prevention, and competitive performance optimization, which aligns closely with the trend toward professionalization and standardization following Breaking’s Olympic inclusion.

### 4.4 Quantitative analysis of the authors

The research team followed the formula proposed by Price (43) to determine the minimum publication threshold for identifying core authors in the field of Breaking competition system research. The formula is expressed as follows:

$$\overset{I}{\underset{m\;+\;I}{\sum }}\;n(x)=\sqrt{N}$$

where N represents the total number of authors and m denotes the minimum number of publications required for an author to be considered a core contributor. The calculation process is as follows:

$$m=0.749\times \sqrt{n_{\max}}\;\approx 1.834$$

**Figure 4:**
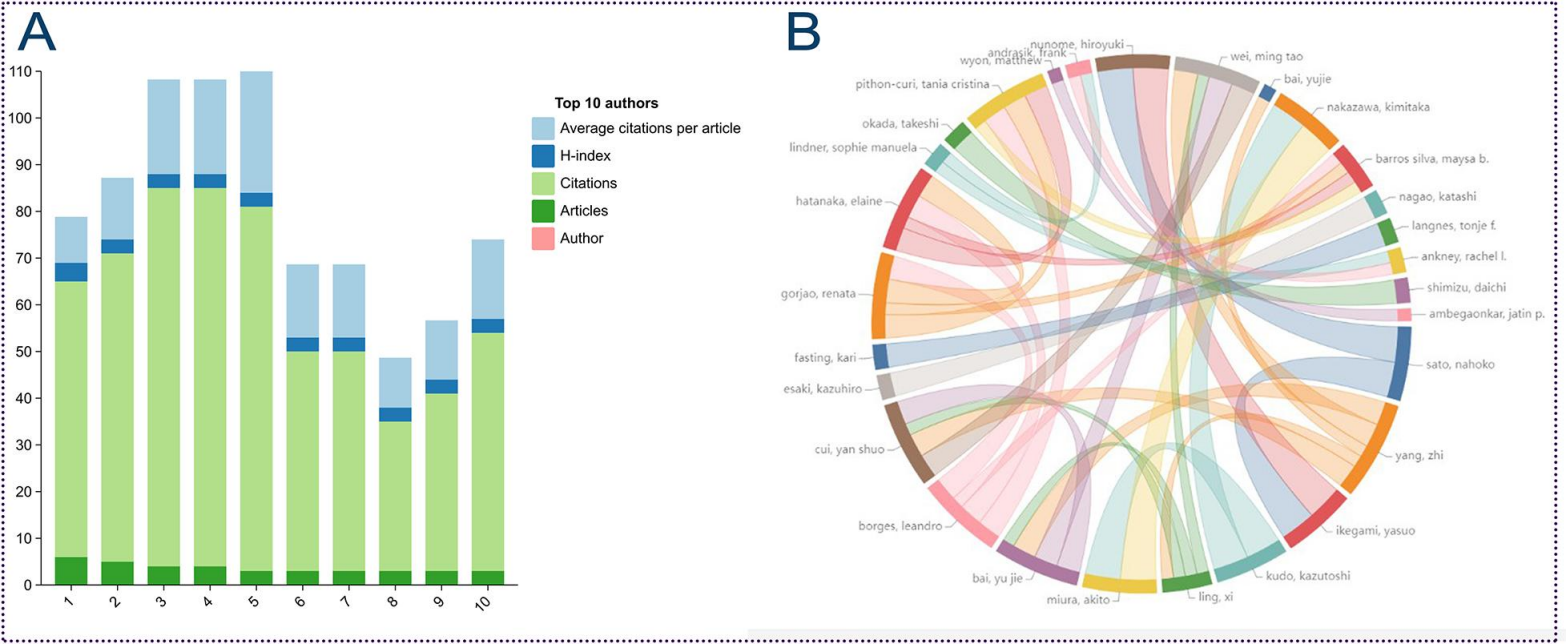
Breaking the top 10 core authors’ collaboration network in the Breaking competition system. **(A)** Academic contributions of the top 10 core authors. **(B)** Visualization of the collaboration network among authors.

Considering that publication counts in bibliometric analysis are discrete integers, this review adopted a rounding-up approach to determine the threshold. Authors with two or more publications (≥2) were therefore identified as core researchers in this field. Table 5 systematically presents the detailed information of the top 10 core authors in the field of Breaking competition system research, while Figure 4 visually illustrates the publication output and citation performance of these core authors.

**Table 5:** Top 10 authors for the Breaking competition system.

Rank	Author	Articles	Citations	H-index	Average citations per article
1	Sato, Nahoko	6	59	4	9.83
2	Wyon, Matthew Alexander	5	66	3	13.2
3	Kudo, Kazutoshi	4	81	3	20.25
4	Miura, Akito	4	81	3	20.25
5	Nakazawa, Kimitaka	3	78	3	26
6	Ikegami, Yasuo	3	47	3	15.67
7	Nunome, Hiroyuki	3	47	3	15.67
8	Yang, Zhi	3	32	3	10.67
9	Zaletel, Petra	3	38	3	12.67
10	Beaulac, Julie	3	51	3	17

The research team conducted an analysis of the academic contributions of core authors. As shown in Figure 4A, the Japanese scholar Sato ranks first in both the number of publications and H-index. Among the core authors, the research group composed of Kudo, Miura, and Nakazawa demonstrates the highest citation impact, indicating the strongest academic influence in this field. This group primarily focuses on whole-body sensorimotor synchronization and dynamic coordination mechanisms in breakdancers (4, 21). In contrast, Sato et al. (22) and Sato and Hopper (44) focuses more on performance evaluation and the establishment of competition scoring criteria. At the same time, Tsiouti and Wyon (27) and Wyon et al. (45) focus on the physiological demands and cardiorespiratory metabolic characteristics of hip-hop dance, providing important physiological insights for the design of physical training programs, performance evaluation, and injury prevention within the Breaking competition system.

Combining Figure 4B and Table 5, several research groups have emerged in the field of Breaking competition systems. Most of these groups were formed after Breaking was included in the Olympics. The most representative example is the collaborative research group led by Yang, comprising members from China, Australia, and Thailand. This group focuses on Breaking evaluation and theoretical development. In addition, the research group led by Vexler concentrates on Breaking training methods and competition judging. Their findings have provided scientific support for the standardization of judging in Olympic Breaking competitions. It is worth noting that the team led by Sato has conducted research spanning both before and after Breaking's inclusion in the Olympics, making it the most enduring research force in the field. The academic foundations established by these research teams have collectively contributed to the rapid development of the Breaking competition system research field.

Overall, the core author structure in the Breaking competition system field demonstrates a multidimensional and specialized division of academic labor, covering research areas such as technical mechanisms and movement science, competition evaluation and rule construction, and physiological demands and physical training. Although the work of these core scholars has strengthened the technical standardization of Breaking, promoted the institutionalization of competition rules, and supported systems for athletic performance and injury prevention, several limitations remain, including insufficient regional representation, structural imbalances in research directions, unclear pathways for practical application, and limited interdisciplinary integration.

### 4.5 Quantitative analysis of journals

Between 1984 and early 2026, a total of 139 academic journals have published research related to the Breaking competition system. Most of the top 10 journals belong to the disciplinary fields of dance studies, medicine, and sports science, as shown in Table 6.

**Table 6:** Top 10 journals for the Breaking competition system.

Rank	Journal	Number of articles	Citations	H-index	Average citations per paper	IF
1	*Journal of Dance Medicine and Science*	8	143	4	17.88	1.6
2	*Frontiers in Psychology*	8	101	5	12.63	2.9
3	*Dance Research Journal*	6	127	5	21.17	0.4
4	*Research in Dance Education*	6	10	2	1.67	0.9
5	*Dance Chronicle*	3	2	2	0.67	0.5
6	*Dance Research*	3	17	1	5.67	0.4
7	*Medical Problems of Performing Artists*	3	50	2	16.67	0.7
8	*PLOS ONE*	3	23	3	7.67	2.6
9	*Applied Sciences*	2	10	2	5	2.5
10	*Frontiers in Sports and Active Living*	2	5	2	2.5	2.6

As shown in Table 6, the *Journal of Dance Medicine and Science* has the highest publication output (eight articles), while the *Dance Research Journal* demonstrates the greatest academic influence, with an average of 21 citations per article. This finding highlights the central role of dance-related journals in the research field of the Breaking competition system.

**Figure 5:**
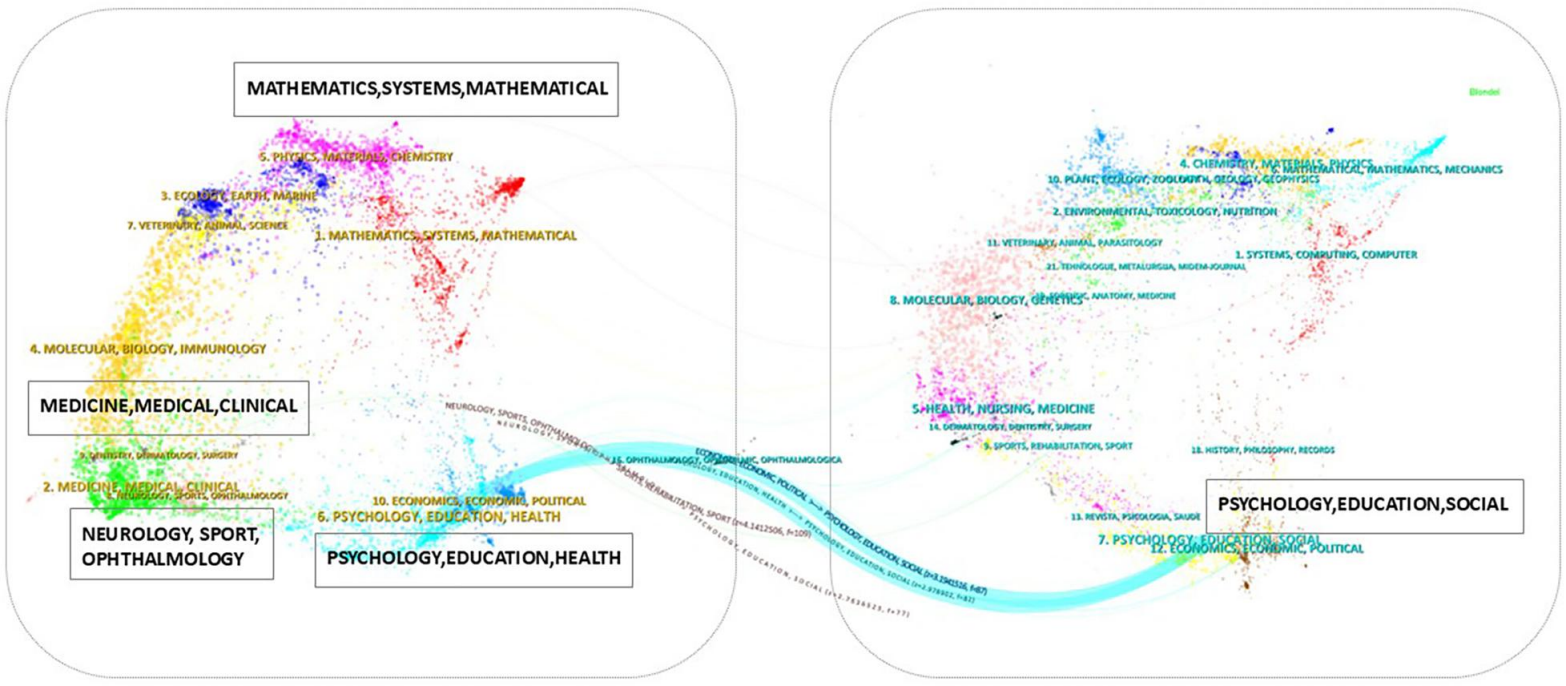
A double map overlay for the Breaking competition system.

To further reveal the citation relationships among journals across different disciplines, this review employed the Dual-map overlays function in the software CiteSpace for analysis (46). As illustrated in Figure 5, the generated map presents citing journals on the left side and cited journals on the right side, while the connecting lines between the two areas visually represent the knowledge interaction relationships at the journal level (47). Using the Z-score algorithm embedded in CiteSpace, clustering analysis was conducted on both citing and cited journals. The results indicate that the field of Breaking competition system research has formed two clear knowledge-flow citation trajectories. Notably, different characteristics in the cited region are distinguished by color variations, and the thickness of citation paths is positively correlated with citation frequency under the Z-score scale (48). Within the cited domain, the field labeled “PSYCHOLOGY, EDUCATION, SOCIAL” contains the largest coverage of records and exhibits a particularly prominent elliptical shape, emphasizing its substantial influence on research concerning the Breaking competition system. The primary citation trajectories originate from this field and extend toward the frontier research areas of “MEDICINE, MEDICAL, CLINICAL” and “PSYCHOLOGY, EDUCATION, HEALTH.”

Furthermore, citation paths from the domain “NEUROLOGY, SPORT, OPHTHALMOLOGY” demonstrate a significant Z-score value (*z* = 4.1412506), highlighting the strong academic influence of this knowledge pathway. In addition, the field “PHYSICS, MATERIALS, CHEMISTRY” also shows notable associations, emerging as one of the representative directions of newly developing interdisciplinary research areas within the Breaking competition system domain.

## 5 Hot topics and evolutionary process

### 5.1 Hot topic frequency and clustering

**Figure 6:**
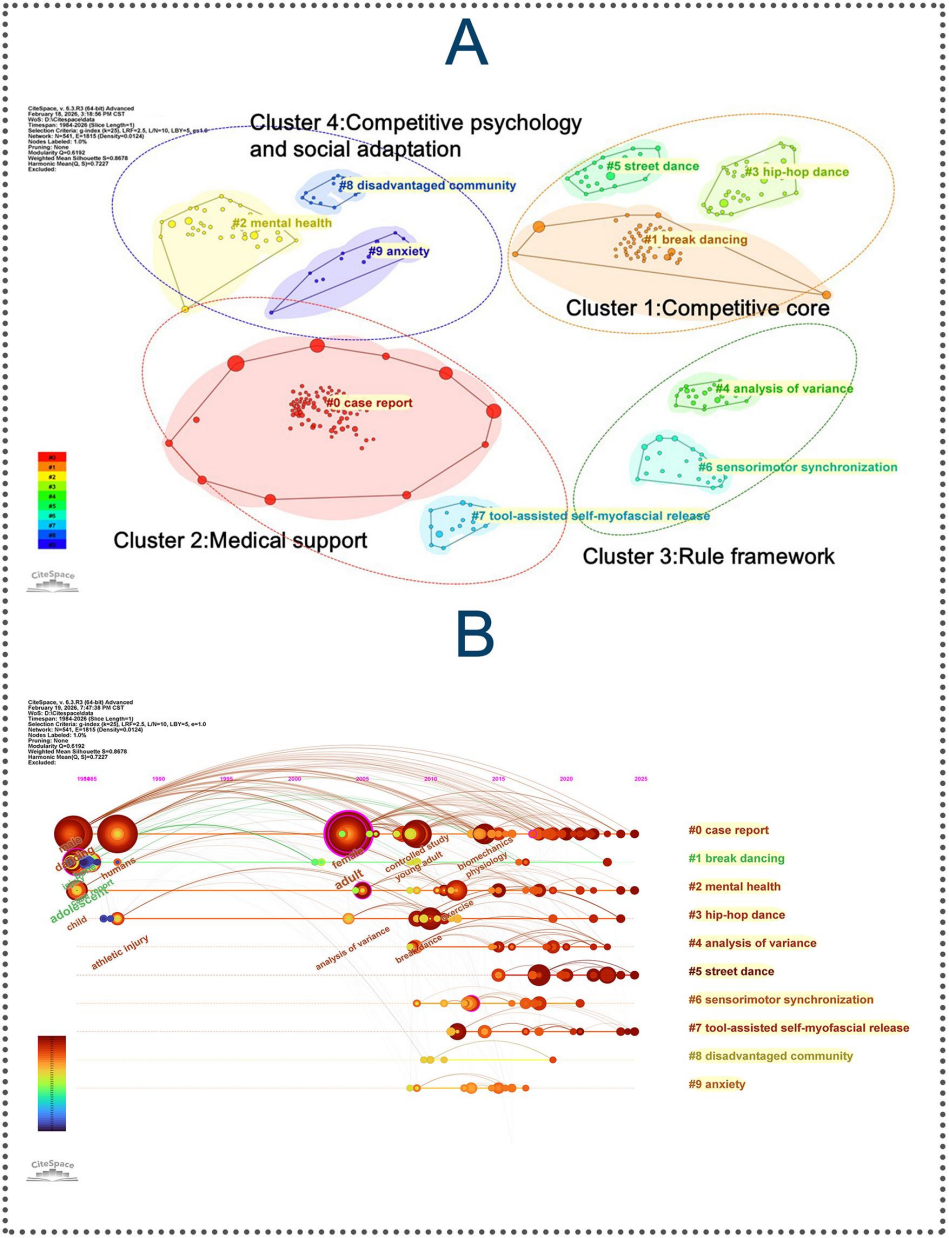
Breaking research trends in competitive systems and changes in hot topics across stages. **(A)** Hotspot clustering. **(B)** Hotspot time zone.

Keywords represent a condensed expression of the core content of research, and analyzing them provides a more intuitive way to identify the major themes and research hotspots within a specific academic field. To comprehensively capture the hotspot keywords in this domain and avoid the omission of key research themes, this review employed the software CiteSpace to conduct an extended keyword analysis based on data from the WoSCC and Scopus databases in the field of Breaking competition system research. The clustering distribution characteristics of hotspot keywords are visually presented in Figure 6A, while the complete list of keywords is provided in Supplementary Document A. As illustrated in Figure 6A, the major research hotspots in the Breaking competition system field can be categorized into four main thematic areas: terminology origins, medical support, rule framework, and competitive psychology and social adaptation.

#### 5.1.1 Theme 1: terminology origins

Theme 1 takes “breakdancing” (an alternative term for Breaking) as the central node, forming a semantic cluster associated with terms such as “street dance” and “hip-hop dance.” Historically, Breaking has been recognized as one of the core technical styles within these street dance forms; however, its terminology has long been debated in academic discourse (19). Consequently, scholars have continuously attempted to clarify the relationship between Breaking and other street dance styles (20, 24). In fact, Breaking was one of the earliest emerging styles among street dances and was later widely referred to as breakdance because of commercial promotion (9). It also constitutes an important component of hip-hop culture (49). However, breakdance is not limited to Breaking alone; the widespread public confusion surrounding the terminology largely stems from commercial marketing practices (50). With the global diffusion of hip-hop culture, numerous new dance styles have developed, gradually situating Breaking and breakdance within the broader category of hip-hop dance. This diversification of styles has even led to inconsistencies in the official terminology used in Olympic promotional contexts, where both “breakdance” and “breaking” appear. Research on the historical origins and conceptual clarification of these terms helps policymakers associated with the International Olympic Committee adopt more precise terminology and contributes to establishing a clearer public understanding of Breaking.

Although recent research has made significant progress in our scientific understanding of injury prevention, most studies still rely on small sample sizes, cross-sectional designs, and laboratory-based assessments. Longitudinal evidence and multicenter validation remain limited, which reduces the generalizability of current research findings. Future research should establish standardized injury monitoring systems and develop evidence-based prevention frameworks applicable to different competitive levels.

#### 5.1.2 Theme 2: medical support

Theme 2 focuses on providing medical support and recommendations for injuries experienced by breakdancers. Breaking has been widely regarded by scholars as one of the most injury-prone dance styles (51, 52). For a long time, breakdancers facing various sports injuries have often failed to seek professional medical assistance, which has led to many athletes prematurely ending their careers (3, 53). To address this issue, numerous scholars have conducted studies on injuries among breakdancers. For instance, one researcher (28) conducted a survey of breakdancers and found that injuries typically occur in the shoulders, arms, and knees, while injured dancers often rely on self-prevention strategies, with only 20% seeking medical treatment. In response to the high injury rate, one researcher (54) carried out a biomechanical study on breakdancers to explain the high incidence of lower-limb injuries in this population. Meanwhile, another researcher (51) called for medical education for breakdancers, including the use of protective equipment, injury prevention and treatment strategies, and biomechanical knowledge. These studies systematically reveal the actual characteristics and key factors contributing to the high risk of injury in this sport, identify core issues such as low rates of medical consultation among dancers and the lack of protective and rehabilitation systems, and promote a shift in the competition system from a purely competitive focus toward a more scientific, safe, and sustainable direction.

Existing research has advanced biomechanical assessment and the evaluation of athletic performance, but there remains significant methodological heterogeneity. Differences in motion capture systems, performance metrics, and analytical methods hinder direct comparisons across studies. Future research should promote standardized biomechanical assessment protocols and enhance ecological validity by incorporating real-world competition settings.

#### 5.1.3 Theme 3: rule framework

Theme 3 focuses on the technical standardization of the Olympic competition system. The keyword cluster “analysis of variance” represents quantitative research methods used to evaluate competitive performance and optimize training programs, while the cluster “sensorimotor synchronization” provides scientific support for Olympic judging indicators such as movement regulation and music synchronization from the perspectives of neuroscience and motor control. In the early stage of competition technical standardization, Sato emerged as one of the most influential pioneers in the field. Sato pointed out the limitations of subjective judgment in Breaking scoring systems and called for the establishment of a reliable evaluation framework (55). Although an increasing number of scholars have echoed this call, and some have attempted to design evaluation models, the research direction has often remained relatively fragmented and lacks a comprehensive framework (56, 57). To address this long-standing issue, Yang and Whatman (36) conducted the first systematic integration of existing qualitative evaluation approaches in Breaking, proposing a comprehensive guideline specifically designed for Breaking assessment. This framework provided practical recommendations for scientifically evaluating athletes and learners and, more importantly, provides a conceptual foundation for designing competition scoring standards. Theme 3 therefore represents the most critical component of Breaking competition system research, driving the field toward standardization and scientific development. Its central contribution lies in establishing a research cycle that moves from problem identification to practical solutions, addressing the core issue of subjective scoring and overcoming the long-standing absence of unified evaluation criteria.

Since the sport was added to the Olympic Games, research on the judging system and scoring standardization in Breaking has increased significantly. However, most studies remain at the descriptive or policy-oriented level, and there is still a lack of empirical validation regarding the reliability of judging, the fairness of scoring, and the consistency of decision-making. Future work should integrate quantitative judging analysis, artificial intelligence, and explainable evaluation models to enhance transparency and objectivity.

#### 5.1.4 Theme 4: competitive psychology and social adaptation

Theme 4 addresses the humanistic and social dimensions of the Olympic competition system. The keyword clusters “mental health” and “anxiety” focus on psychological regulation and intervention for athletes in competitive environments, ensuring both competition fairness and athlete wellbeing. The cluster “disadvantaged community” reflects the social origins of Breaking and provides insights into its cultural dissemination and social value realization within the Olympic framework. Beyond physical injuries, mental health is another critical issue that should not be overlooked (58). As a newly introduced Olympic sport, Breaking requires athletes to constantly prepare for uncertain challenges, which significantly increases psychological pressure. Lai et al. (23) examined how dance participation enhances wellbeing and self-efficacy among dancers, while Hall et al. (26) investigated the mechanisms of training-induced hair loss among breakdancers, offering preventive recommendations and contributing to the broader effort of improving breakdancers' wellbeing.

In summary, the four themes outlined above collectively depict the knowledge structure of Breaking competition system research. Studies on terminology origins provide the conceptual foundation for disciplinary definition and discourse standardization; medical support research ensures athlete health protection and sustainable development; rule framework studies determine the level of standardization and scientific rigor within the Olympic competition system; and competitive psychology and social adaptation research defines the humanistic values and social adaptability of the sport.

### 5.2 Evolutionary trends analysis

Figure 6B presents the evolutionary characteristics of research on the Breaking competition system, visually reflecting the differences in the frequency and distribution of keywords across different stages. This visualization helps identify and analyze the dynamic transformation of research themes within the field (59). Taking 2018, when the International Olympic Committee officially announced the inclusion of Breaking in the Olympic Games, as the critical turning point, the research team divided the evolution of the Breaking competition system into three phases.

#### 5.2.1 Phase one (January 1984–December 1999): Spontaneous research phase

The period from early 1984 to the end of 1999 represents the initial stage before Breaking entered the Olympics. Influenced by media publicity, Breaking entered the public’s field of vision, but the public had little understanding of this culture (60). Descriptions of Breaking mainly focused on the background of Breaking culture and the symptoms caused by breakdance (61). In these studies, most only described that Breaking became a popular culture worldwide and did not deeply discuss its causes and mechanisms (62, 63). In addition, many articles lacked DOI number or were not indexed, resulting in very limited research in this period (24).

#### 5.2.2 Phase two (January 2000–December 2017): Initial development phase

After entering the new millennium, the number of Breaking studies began to increase gradually. In this stage, “physiology,” “biomechanics,” and “exercise” were hot keywords, and related studies focused on three core contents: analysis of movement mechanisms, identification of injury risks, and optimization of sports performance in Breaking, forming a research orientation supported by empirical data for competitive development. At the level of competition standards, movement characteristic indicators proposed by Sato et al. (64) provided objective evaluation references for the scoring system for the first time, making up for the deficiency of traditional competitions relying only on subjective scoring. At the level of athlete protection, some researchers (28, 51) focused on injury research, clarified the physical differences of breakdancers at different levels, promoted the formation of graded protection concepts in the competition medical support system, and provided reference for subsequent protective equipment development and injury emergency plan formulation. At the level of training scientificization, Miura et al. (65) revealed the differences in whole-body sensorimotor synchronization between professional and non-professional breakdancers, emphasized the coordination advantages of professional breakdancers, and provided a reproducible research paradigm for the cultivation of basic abilities of breakdancers. However, these studies still have several important limitations: in terms of research depth, existing biomechanical studies mostly focus on local analysis of single movements (such as coordination between fingers and beats) (21), lacking systematic biomechanical evaluation of complete Breaking routines (such as the connection process of toprock, footwork, and power move), making it difficult to fully support the quantitative design of scoring dimensions such as movement continuity and music adaptation in competitions. In terms of research application, although injury prevention studies have clarified risk factors (54), they have not combined biomechanical findings with specific training intervention programs, resulting in breakdancers understanding the causes of injuries but being unable to effectively prevent them. In terms of research breadth, studies on physiological differences of Breaking dancers with different age groups and training backgrounds are almost blank, making it difficult to meet the needs of graded training and personalized training in the Olympic competition system. These shortcomings have also become the core directions that need to be broken through in subsequent research.

#### 5.2.3 Phase three (January 2018–February 2026): Olympic-oriented standardization construction phase

With Breaking being identified as an Olympic competitive event by IOC (1), the research field of its competition system has formed a multidimensional and cross-field focus pattern, covering six core areas: Olympic rule adaptation, judging optimization, teaching and training reform, cultural inheritance protection, physiological support upgrading, and terminology standardization, and each field has different research teams participating, reflecting the comprehensiveness and diversity of research.

(1) In the field of Olympic rule adaptation, a researcher (36), in response to the rapid increase of qualitative research after Breaking entered the Olympics, developed for the first time an evaluation standard suitable for Olympic Breaking research, solving the problem of mismatch between general research standards and Olympic project characteristics; Wei et al. (24), combined with the needs of the 2024 Summer Olympics, proposed a formal formative assessment framework in Breaking teaching and clarified the application path of this framework in the training of Olympic athlete echelon; another researcher (42), based on semiotic theory, analyzed the three-dimensional evolution of body posture and competition form before and after Breaking entered the Olympics, providing theoretical support for balancing cultural attributes and competitive attributes in Olympic events.
(2) In terms of judging system research, a researcher (55), through an empirical analysis of Japanese hip-hop dance competitions, pointed out systematic biases such as undefined technical difficulty and unexplained scoring levels and proposed that a three-in-one scoring framework, namely, dimension definition, movement standards, and level interpretation, should be established; another researcher (41) identified key influencing variables of judges' scoring through quantitative research and suggested that judge qualification certification should break through experience discrimination and construct a personalized curriculum system including cross-cultural adaptation content.
(3) In the field of teaching and training, a researcher (66) explored integrating Breaking elements into daily teaching to improve students' learning efficiency, providing a new path for the enlightenment education of Olympic reserve talents; another researcher (7) updated and expanded the definition of hip-hop pedagogy and clarified the application boundary of Breaking in formal education environments; another researcher (67), through comparative experiments, confirmed that Breaking-style headstand practice (on hard ground) had a significantly lower fall rate than traditional gymnastics mat practice, providing a safety optimization scheme for basic movement training of Olympic athletes.
(4) In the field of cultural symbol inheritance, a researcher (68), taking the Ecuadorian Naturalz dance group as the research object, pointed out that after Breaking entered the Olympics, it is still necessary to retain the utopian ideal of affirming street values to avoid the loss of cultural essence caused by competition.
(5) In the field of physiological support, a researcher (45), through cardiopulmonary function tests, found that Breaking has higher cardiopulmonary demand and blood lactate levels compared with new style hip-hop dance, providing a physiological basis for targeted physical training of Olympic athletes.
(6) In the field of terminology standardization, a researcher (19) designed a street dance knowledge system and clarified the terminology definition of Breaking among multiple dance types; another researcher (20), focusing on the Youth Olympic Breaking event, clarified the confusion between Breaking and breakdance, providing a reference for the unification of terminology in Olympic official promotion.

Overall, Phase three has produced multidimensional positive impacts on the research of Breaking competition system construction. For example, it has improved the completeness and systematicity of the field knowledge system. Through the collaborative research of six core areas, it has filled key gaps such as rule standards, Olympic adaptation, and cultural balance before entering the Olympics. Another example is that it has enhanced the practical transformation value and international recognition of research results, improved the academic status of this field in sports science, and attracted more interdisciplinary forces to participate. The most prominent point is that this stage has constructed a closed-loop development model of research, practice, and competition. Research is no longer limited to the theoretical level but is continuously optimized through feedback from Olympic events, providing sustainable momentum for the long-term development of the Breaking competition system.

### 5.3 Critical evaluation of existing research

Although research on the competitive system of Breaking has expanded rapidly in recent years, several methodological limitations persist in the existing literature. First, many studies employ cross-sectional or descriptive research designs, which limit the ability to establish causal relationships or assess long-term trends. Second, there is significant heterogeneity among research methods, measurement indicators, and analytical frameworks, making comparisons across studies difficult and weakening the cumulative strength of the evidence. Third, a large portion of the current literature focuses on technical performance, biomechanics, and injury prevention, while attention to reliability assessments, governance mechanisms, institutional development, and policy evaluation remains relatively limited. Furthermore, empirical research on elite competitions remains scarce, and many of the proposed competition models have not yet been validated in real-world competitive settings.

These findings suggest that future research should shift from descriptive surveys toward more rigorous longitudinal studies, interdisciplinary research, and evidence-based research. To establish a scientifically sound, culturally sensitive, and internationally standardized Breaking competition system, greater collaboration among sports scientists, governing bodies, referees, coaches, and cultural scholars will be essential.

## 6 Frontier development trends

### 6.1 Hotspot evolution analysis

**Figure 7:**
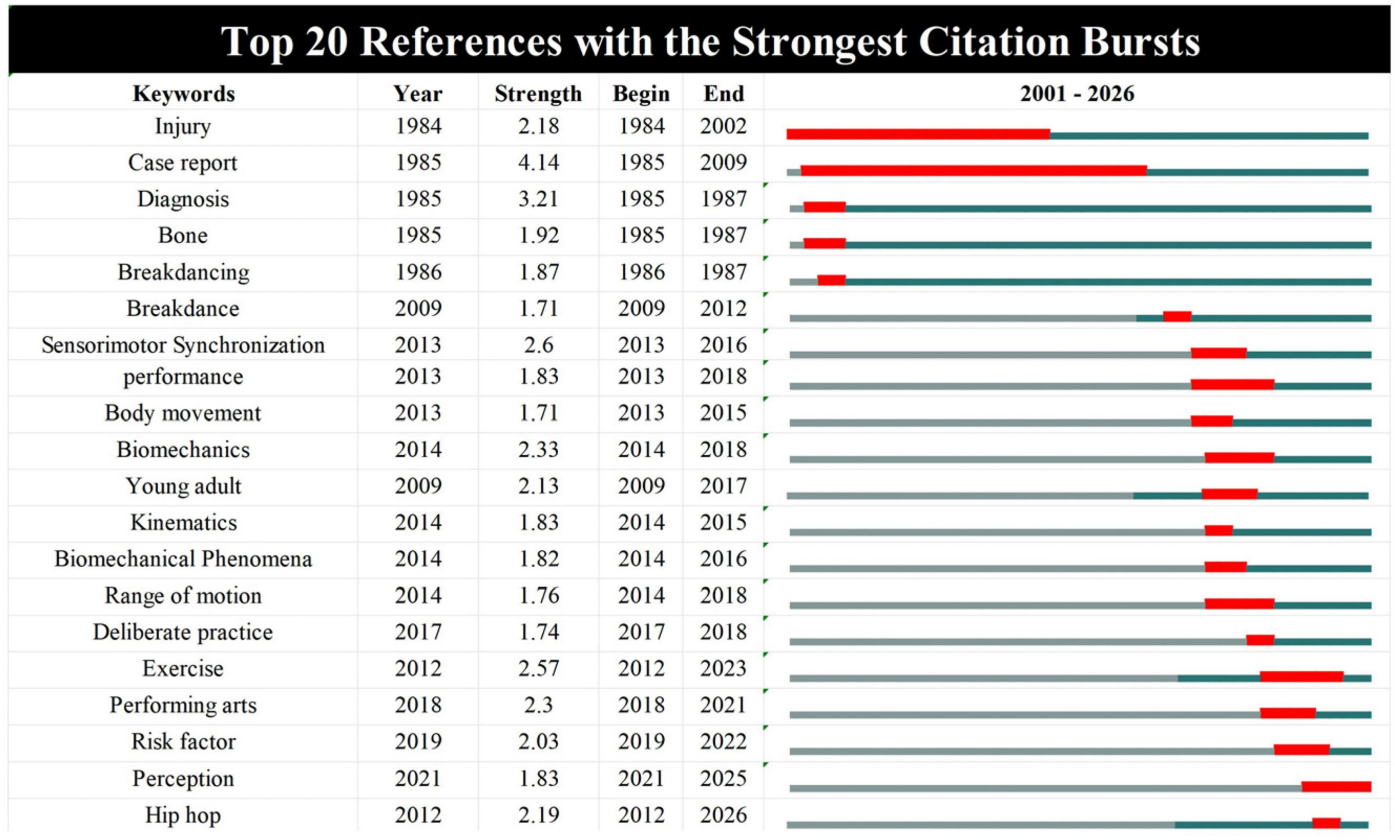
Top 20 keywords with the strongest citation bursts.

Burst keywords can intuitively reflect the evolution trend of hotspots in a specific research field. This review, with the help of the function of Burst detection in CiteSpace, screened and presented the top ten keywords in terms of burst intensity (see Figure 7 for details). Figure 7, based on the burst detection algorithm, sorts out the top 20 keywords in terms of burst degree, in which the red-marked period represents the high citation attribute and burst intensity of keywords in the corresponding years. With the help of the above burst keywords, it is possible to effectively identify the core hotspots in different research stages and the evolution law of research directions (30).

As shown in Figure 7, the field of the Breaking competition system presents obvious evolutionary stages. Since 1984, “Injury” first burst, indicating that a small number of scholars had already noticed the high incidence of injuries in Breaking and emphasized the reminder of sports injuries among breakdancers. In the same period, “Case report,” “Diagnosis,” “Bone,” and “Breakdancing” subsequently appeared, confirming this trend, that is, Breaking injuries were no longer limited to a small range, but a problem that needs to attract high attention from the breakdancer group. However, between 2009 and 2018, “Sensorimotor Synchronization,” “Body movement,” “Biomechanics,” “Kinematics,” and “Biomechanical Phenomena” indicate that researchers have shifted from an early simple description of injury phenomena and epidemiological statistics to a scientific and quantitative exploration of breakdancer movement control mechanisms, sports biomechanical characteristics, sensorimotor coordination ability, and kinematic laws of movements. This phenomenon indicates that the sports science research of Breaking has shifted from a passive postevent description of injuries to an active scientific analysis of movement mechanisms and sports performance, marking that this field has moved from empirical observation to a mature stage of empirical, quantitative, and mechanistic research, and also laid a key theoretical and methodological foundation for subsequent Olympic-oriented technical standardization, training scientificization, and scoring objectification. After entering 2018, “performing arts,” “risk factor,” “perception,” and “hip hop” became hotspot words, which centrally reflect that under the background of entering the Olympics, Breaking research has shifted from a single analysis of sports biomechanics and movement mechanisms to a multidimensional comprehensive exploration of performing arts attributes, injury risk factors, subjective perception and evaluation systems, and the origin of hip hop culture, marking that the research scope has expanded from pure sports science to the direction of integration of art, health, cognition, and culture, and overall presents a systematic development trend that is more in line with Olympic competition needs and takes into account humanistic attributes and practical support. It is worth noting that “hip hop” has become the hotspot word with the longest continuous popularity since the millennium, which fully shows that Breaking has always been rooted in the parent body of hip hop culture, and its process of competition, standardization, and Olympization has never been separated from the cultural origin, and also confirms that research in this field always needs to maintain a balance among sports competition, artistic performance, and street culture. Cultural attributes are not only the core feature that distinguishes Breaking from other Olympic events, but also the underlying logic and stable core that runs through the whole process of its academic research development.

In summary, research related to the Breaking competition system has always revolved around four main lines: safety guarantee, technical scientificization, competition standardization, and cultural subjectivity. Overall, it presents common characteristics of shifting from passive response to active construction and from a single dimension to a multidimensional integration, providing continuous theoretical supply and directional guidance for the framework construction, rule improvement, and practical implementation of the competition system. However, at the same time, existing research also has obvious limitations: the themes have long been in a relatively independent and scattered state, the degree of cross-field integration is insufficient, and a unified theoretical and practical closed loop supporting the efficient operation of the competition system has not been formed; the research focus mostly stays at the level of phenomenon description, mechanism analysis, and conceptual discussion, and there are still relatively few practical and implementable results such as rule design, scoring quantification, judging consistency, and cross-cultural adaptation oriented to Olympic competition scenarios; the coordination mechanism between cultural value and competitive standards has not yet been truly established, resulting in an emphasis on balance at the theoretical level, while in practice it still tends toward competition and standardization, making it difficult to fully take into account the cultural uniqueness of the project and the fairness and professionalism of the competition.

### 6.2 Future development trends of the Breaking competition system

**Figure 8:**
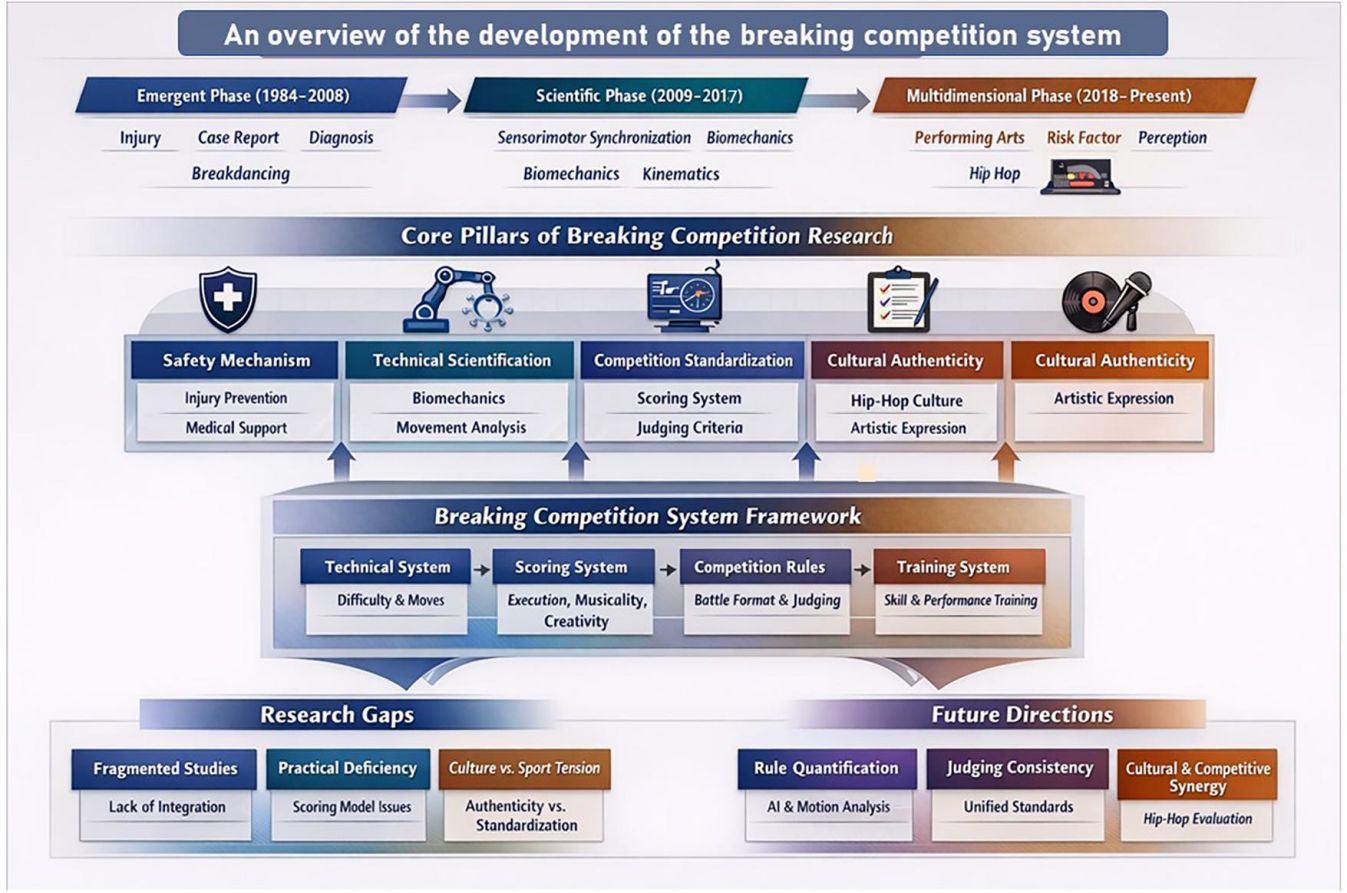
An overview of the development of the Breaking competition system.

We summarized the research from 1984 to 2026 and designed a relationship diagram of the development mechanism of the Breaking competition system, as shown in Figure 8.

As shown in Figure 8, the research field of the Breaking competition system can be explained from five levels: hotspot evolution, core research pillars, competition system framework, research gaps, and future development paths. This field has experienced a development trajectory from early empirical observation to multidisciplinary integration and further points out the key development directions for the construction of the Breaking competition system.

Through a hotspot evolution analysis, it is found that the research field of Breaking competition system construction shows a clear evolutionary trajectory from early problem awareness to systematic theoretical construction. In this context, the content framework of this field can be understood through four dimensions, namely technical system, scoring system, competition rules, and training system. However, current research is still limited by the fragmentation of disciplinary methods, insufficient practical research on scoring quantification and judging consistency, and the persistent tension between cultural authenticity and competition standardization. Therefore, future development needs to seek a balance between sports competitiveness and the essence of hip-hop culture. Based on the above research foundation and practical problems, researchers further propose three important directions for the future development of the Breaking competition system.

(1) Rule quantification. By introducing artificial intelligence, motion recognition technology, and sports data analysis, objectively quantify Breaking technical movements and competition performance, thereby improving the scientificity and transparency of the scoring system.
(2) Judging consistency. Future research needs to strengthen the study of judging reliability and improve the consistency among different judges by unifying scoring standards and judge training systems.
(3) Cultural and competitive synergy. In the process of developing the competition system, it is necessary to establish an evaluation mechanism that takes into account both competitive standards and cultural expression, so that hip-hop cultural values and competitive sports norms can achieve coordinated development.

Overall, the development trend of Breaking competition system construction shows the general characteristics of transformation from single-problem focus to systematic construction, from empirical observation to scientific analysis, and from disciplinary dispersion to multidimensional integration. In this process, safety guarantee, technical scientificization, competition standardization, and cultural subjectivity jointly constitute the core pillars of the development of the Breaking competition system, and future research needs to further strengthen theoretical integration and practical application, so as to promote a more stable and sustainable development of the Breaking competition system between global competitive sports and hip-hop culture.

## 7 Theoretical interpretation of the evolution of the Breaking competition system

The findings of this bibliometric study can be further interpreted through a variety of complementary theoretical perspectives, which help explain the transformation process from grassroots cultural practices to internationally recognized competitive sports.

From the perspective of the sociology of sport, the development of Breaking reflects the process by which an emerging sports culture gradually gains social legitimacy, organizational recognition, and institutional support. With the inclusion of Breaking in the Olympic Games, the number of related publications has increased rapidly, indicating a growing interaction among cultural identity, sports participation, and institutional development. From the perspective of institutionalization theory, the development of the Breaking competition system is a gradual process involving the institutionalization of rules, the standardization of organizations, and the consolidation of governance. The evolution from informal street matches to internationally standardized competitions requires the establishment of unified judging criteria, standardized competition procedures, athlete eligibility regulations, and governance mechanisms that enhance the legitimacy and sustainability of Breaking competition. From the perspective of cultural studies, although Breaking has become increasingly institutionalized, it remains deeply rooted in hip-hop culture. The coexistence of cultural expression, creativity, authenticity, and competitive standardization reflects a dynamic balancing act between preserving cultural identity and adapting to the demands of elite sports organizations. This balance has become one of the defining characteristics of contemporary Breaking research. From the perspective of Olympic studies and sports governance, the inclusion of Breaking in the Olympic Games has accelerated international cooperation, policymaking, judging system reforms, and scientific research. Governing bodies increasingly emphasize evidence-based decision-making, athlete wellbeing, transparent judging systems, and standardized event management. Consequently, research in recent years has gradually shifted from descriptive cultural discussions toward interdisciplinary studies integrating biomechanics, injury prevention, psychology, governance mechanisms, and performance evaluation.

Taken together, these theoretical perspectives provide a broader interpretive framework for understanding the evolution of themes identified in this bibliometric analysis. The observed shifts in themes do not represent isolated research topics but rather reflect the institutional maturation, cultural negotiations, and shifts in governance models that the sport of Breaking has undergone as it developed into a globally recognized competitive sport.

## 8 Olympicization and the tension between cultural authenticity and sport institutionalization

As a practice rooted in hip-hop culture, Breaking has traditionally emphasized creativity, improvisation, community involvement, personal expression, and resistance to excessive institutional control. Increasingly standardized judging criteria and competition formats may inadvertently restrict artistic freedom, undermine stylistic diversity, and reshape the cultural essence that originally distinguished Breaking from traditional competitive sports. Therefore, the evolution of the Breaking competition system should not be understood as a process in which culture is gradually replaced by sport, but rather as an ongoing negotiation between cultural authenticity and institutional legitimacy. Maintaining this balance is crucial to the sustainable development of Breaking. Future policymaking should be dedicated to upholding the core values of hip-hop culture, including originality, creativity, individuality, and community participation. At the same time, it should promote transparent governance, fair judging, athlete welfare, and international competitive standards. This comprehensive approach will enable the sport of Breaking to continue developing into a globally recognized competitive discipline, while preserving its cultural identity.

## 9 Conclusions, limitations, and prospects

### 9.1 Conclusions

This review analyzed publications on the Breaking competition system retrieved from the WoSCC and Scopus between 1984 and 2026 using bibliometric methods. Through bibliometric methods, thematic clustering, temporal evolution, and content mining analysis were carried out, and it was found that the research in the field of Breaking competition system shows the following characteristics:

#### 9.1.1 Main publications and research forces

The Breaking competition system has become an important research direction after Breaking was included in the Olympic Games, which is related to the sustainable development of Breaking competition. At present, the research forces for the construction of the global Breaking competition system are unevenly distributed and highly concentrated in a few countries. The United States, as the birthplace of Breaking, has the largest number of published articles. In addition to the United States, China, Canada, South Korea, Australia, and other countries have paid attention to Breaking as a new Olympic event. In terms of international cooperation, a pattern has been formed with China as the hub, led by the United States and the United Kingdom, and supplemented by other Asian countries, showing obvious regional cluster characteristics.

Two groups of core authors appeared before and after Breaking entered the Olympics. Before entering the Olympics, the research group composed of Miura, Kudo, Nakazawa, and others was the most prominent, focusing on research on the athletic ability of breakdancers. After entering the Olympics, a group of emerging scholars appeared, including Matthew Wyon, Zhi Yang, and Vexler, focusing on research on the physiological functions, teaching evaluation, and competition scoring of breakdancers. Among these scholars, Sato is the scholar who has continuously produced results before and after Breaking entered the Olympics, maintaining a high level of attention to competition scoring and performance evaluation research. In terms of cooperation, the most typical collaborative team comes from China, Australia, and Thailand. The research group led by Yang focuses on research in the field of Breaking evaluation.

In terms of institutions, the one with the largest number of publications is The University of Tokyo, and the most influential is the Japan Society for the Promotion of Science, showing the core position of Japanese research institutions in the field of Breaking competition system research.

In terms of journals, *Journal of Dance Medicine and Science* ranks highest in the number of publications, and *Dance Research Journal* shows the greatest influence. This indicates that articles related to Breaking competition system research are mainly published in dance journals. In addition, among the research fields of journals carrying research related to this review, “MEDICINE, MEDICAL, CLINICAL” and “PSYCHOLOGY, EDUCATION, HEALTH” occupy the frontier of research fields, and “NEUROLOGY, SPORT, OPHTHALMOLOGY” have become the most influential research paths.

#### 9.1.2 Evolution trends and focus areas of the Breaking competition system

Through a time evolution analysis of high-frequency keywords, the research field of the Breaking competition system has experienced three stages of evolution, namely spontaneous research phase, initial development phase, and Olympic-oriented standardization construction phase.

(1) Spontaneous research phase focused on discussing the background of the emergence of Breaking culture and injuries caused by breakdance.
(2) Initial development phase focused on the analysis of movement mechanisms, identification of injury risks, and optimization of sports performance in Breaking.
(3) Olympic-oriented standardization construction phase focused on Olympic rule adaptation, judging optimization, teaching and training reform, cultural inheritance protection, physiological support upgrading, and terminology standardization.

Through a high-frequency keyword clustering analysis, four hotspot clusters are obtained: terminology origins, medical support, rule framework, and competitive psychology and social adaptation. Among them, the four clusters provide support for the Olympic competition system of Breaking from four aspects: cultural origin, medical support, competition rules, and social adaptation. Terminology origins provides a theoretical foundation, medical support ensures the training safety of athletes, rule framework provides reference for rule formulation, and competitive psychology and social adaptation define the humanistic value and social adaptability of the project development. These clusters construct a complete chain for the sustainable development of the Breaking competition system.

In summary, the development of the Breaking competition system field showed a development status from scattered spontaneous exploration to systematic and standardized construction, from single-problem analysis to multidimensional coordinated integration, and from empirical description to scientific empirical support. The development momentum continues to improve, the research boundaries continue to expand, and the theoretical depth and practical application value continue to increase, and the field as a whole has entered a stage of rapid maturity that closely aligns with the needs of Olympic competition.

#### 9.1.3 Evolution of research frontiers in the Breaking competition system

From the results of burst keyword analysis, “Injury” and “Case report” have the longest duration of continuous bursts, and most burst keywords mainly began in 2009, marking that the field entered a period of rapid expansion after the transformation to scientificization and systematization. The research focus quickly shifted from early exploration of breakdancer culture and injuries to training optimization and competition scoring design. “Hip hop,” as the most frontier hotspot, indicates that cultural attributes are the underlying logic and stable core that runs through the whole process of Breaking academic research development. Looking ahead, the research field of the Breaking competition system will develop in depth toward three areas: rule quantification, judging consistency, and cultural and competitive synergy.

### 9.2 Strengths and limitations

A key strength of this review is the integration of two multidisciplinary databases (WoSCC and Scopus) with various bibliometric tools to provide a comprehensive overview of the evolution of the Breaking competition system. Although this review provides a comprehensive overview of the research developments regarding the Breaking competition system, there are several limitations that should be noted. While WoSCC and Scopus are widely regarded as the two most comprehensive interdisciplinary citation databases in bibliometric research, publications available only in other databases—such as PubMed, EBSCO, Google Scholar, or regional databases—were not included. Consequently, some relevant studies may have been omitted, which could lead to database selection bias. Second, the analysis included only English-language publications. Since Breaking originated in a multicultural context and has developed globally, important research published in other languages may have been excluded, thereby introducing a language bias and limiting the representativeness of regional studies. Third, bibliometric indicators are inherently influenced by citation-related factors, including publication year, disciplinary citation practices, journal visibility, and self-citations. Consequently, publications with high citation counts do not necessarily represent the highest methodological quality or scientific impact, while recently published studies may be at a disadvantage due to insufficient time for citations to accumulate. Fourth, although the search strategy aimed to comprehensively cover studies related to the Breaking competition system, some research from cultural studies, dance studies, performance studies, or related interdisciplinary fields may not have been retrieved because of differences in database coverage, indexing methods, or terminology. Future research could incorporate databases from additional disciplines to achieve broader interdisciplinary coverage. Furthermore, a bibliometric analysis primarily reveals publication patterns, collaboration, citation trends, and thematic evolution, rather than directly assessing the methodological quality, level of evidence, or practical impact of individual studies. Therefore, future research should combine bibliometric methods with systematic reviews, qualitative analysis, or meta-analyses to provide a more comprehensive assessment of the development of the Breaking competition system.
